# Relationship of Sodium Intake with Overweight/Obesity among Chinese Children and Adolescents: Data from the CNNHS 2010–2012

**DOI:** 10.3390/ijerph18094551

**Published:** 2021-04-25

**Authors:** Kehong Fang, Yuna He, Yuehui Fang, Yiyao Lian

**Affiliations:** National Institute for Nutrition and Health, Chinese Center for Disease Control and Prevention, No. 29 Nanwei Road, Xicheng District, Beijing 100050, China; kehongsky@163.com (K.F.); fangyh@ninh.chinacdc.cn (Y.F.); lianyy@ninh.chinacdc.cn (Y.L.)

**Keywords:** sodium, sodium density, overweight, obesity, Chinese children and adolescent

## Abstract

This study aims to examine association between sodium intake and overweight/obesity among Chinese children and adolescents. Data were obtained from China National Nutrition and Health Surveillance (CNNHS), 2010–2012. All participants recruited in this study aged 7–18 years old and provided complete dietary data on three-day consecutive 24 h dietary recalls combining with the household weighing method. Body Mass Index (BMI) was used to define overweight/obesity, and waist-to-height ratio (WHtR) was used to define abdominal obesity. Sodium intake showed association with risk of overweight/obesity assessed by BMI in the highest tertile group with OR of 1.48 (95%CI 1.13–1.94) and 1.89 (95%CI 1.33–2.67) for WHtR. After adjusted for gender, age, household income, area, energy, carbohydrates, protein, fat, saturated fatty acids, and fiber intake, the relationship between sodium intake and overweight/obesity and abdominal obesity are not changed. The same results were founded in subjects aged 10–18 years old. Our results reveal a positive association between sodium intake and overweight/obesity in Chinese children and adolescents, independent of energy consumption.

## 1. Introduction

Over the past several decades, the rate of obesity among children and adolescents has been increasing rapidly. The World Health Organization (WHO) reports that more than 124 million children and adolescents (6% of girls and 8% of boys) were obese in 2016, and half of them lived in Asia [1]. With the rapid change of living lifestyles in China, the prevalence rate of overweight has raised to 15.1% and obesity has raised to 10.7% in 2017 for children and adolescents [2,3]. Early development of overweight/obesity can persist throughout childhood and even affect weight in adulthood, and evidence shows that obese children and adolescents are at increased risk of hypertension, diabetes, metabolic syndrome, cardiovascular disease, and cancer in adulthood [4,5,6]. Preventing obesity in children and adolescents has become one of the important public health issues of concern in many countries [7,8].

The root cause of overweight/obesity is the imbalance of energy intake and expenditure, and the relationship between poor healthy lifestyles, such as high-energy diets and poor physical activity, and obesity has been verified [7]. In recent years, studies have continuously shown that a high-sodium diet will increase the risk of overweight/obesity [8,9,10,11,12]. However, the research results about the effect of high-sodium diet on overweight/obesity are not consistent. Previous studies have suggested that the increased risk of overweight/obesity due to the increased sodium intake along with the increased of energy intake and sugary beverages [13,14]. Nevertheless, recent study suggested that there may be an association between sodium intake and obesity among children and adolescent, independent of energy intake [15,16]. There are also evidences regarding sodium intake in relation to body weight in zoological studies [17]. On the contrary, one study demonstrated that there is no relationship between sodium intake and overweight/obesity [10]. In addition, there are few studies have concentrated on the effect of macronutrients on the relationship between dietary sodium and overweight/obesity. 

Thus, whether sodium intake is an independent risk factor for overweight/obesity is unknown, and few studies concentrate on the effect of macronutrient intake on association between sodium intake and overweight/obesity. Therefore, present study we explored the association between sodium intake and overweight/obesity among Chinese children and adolescents, and determined whether this association was independent of energy and macronutrient intake.

## 2. Materials and Methods

### 2.1. Study Population

The study was based on data from the China National Nutrition and Health Survey (CNNHS) 2010–2012, which was a nationally representative survey done by the Chinese Center for Disease Control and Prevention. The CNNHS 2010–2012 was carried out on stratified multistage systematic clustered random sampling method with proportional to the population to form a representative sample of China as a whole, including 150 survey site, and the design and methods of CNNHS 2010–2012 had been described in details previously [18]. In brief, a total of 3300 children and adolescents aged 7–18-year-old with food data and anthropometric measurements data were recruited in this study. The survey was approved by the Ethical Committee of the National Institute for Nutrition and Food Safety of the Chinese Center for Disease Control and Prevention (2013(018)) [19].

### 2.2. Estimating Nutrients Intake

The data were collected by trained and certified staff using standardized procedures [19]. The questionnaire was designed to collect information about socio-demographics, diet and where to eat. Dietary data were collected through three consecutive days of 24 h recalls for individual (including two weekdays and one weekend day), combined with a household food weighting method over these days. The amount of edible oil and ingredients (such as salt, soy sauce, chicken essence, and other condiments) used at home were measured by a uniformly calibrated electronic scale. If the younger subjects were unable to provide the information by themselves, the person who took care of them to complete the dietary survey. Dietary sodium intake from each food was calculated according to the Chinese Food Composition Table [20], and the food not included in the table was grouped into the most similar categories. Sodium contributed by food from eating outside was calculated according to consecutive days of 24 h recalls and the Chinese Food Composition Table. In order to estimate the sodium intake contributed by edible oil and ingredients of eating outside, the sodium intake of each participant was estimated according to the share of energy intake from eating outside and eating at home. Eating outside defined as food prepared at restaurants, including fast-food restaurants, full-service restaurant, and outdoor fixed food stalls [21].

Protein, fat, saturated fatty acids, carbohydrates, fiber, and energy intake were estimated just like dietary sodium intake. The sodium density was used to reflect how much dietary sodium intake regardless of daily energy intake. In this study, sodium density (mg/kcal) was defined as the ratio of daily dietary sodium intake (mg/day) to daily energy intake (kcal/day). 

### 2.3. Anthropometric Measurements

All subjects were concentrated in community health center and their height, weight and waist circumference were measured by trained research staff following standard protocols. Waist circumference (WC) was measured at the narrowest area between the lowest rib and the uppermost lateral border of the right iliac crest. Height and WC were registered to the nearest 0.5 cm, weight was registered to the nearest 0.1 kg. Body mass index (BMI) (kg/m^2^) was calculated as the body weight (kg) divided by height (m) squared, and overweight/obesity were defined using the WHO standard [22], abdominal obesity was defined as waist-to-height ratio (WHtR) of more than 0.5 [23].

### 2.4. Statistical Analysis

All analyses were carried out with SAS 9.4 (SAS Institute, Cary, NC, USA). Applying the post-stratification population sampling weights derived for the dietary surveys from the sampling probability of the 2010 Chinese population (based on census data). The differences between groups were analyzed using survey means or weighted percentages for survey design. The Kruskal–Wallis test was used to analysis continuous variables with skewed distribution. A survey logistic regression was used to test the associations between tertile of sodium intake or sodium density and overweight/obesity to calculate the crude and multivariate-adjusted OR along with 95% CI for each tertile compared with the lowest tertile. In total, three multivariate models were tested to explore the roles of sodium intake in relation to adiposity. Model 2 adjusted for age, sex, household income and area, Model 3 adjusted for age, sex, household income, area, energy intake. Model 4, additionally, adjusted for protein and fats, in addition to all the mentioned variables in Model 3. A *p* value < 0.05 was considered to be statistically significant. 

## 3. Results

### 3.1. Baseline Characteristics of the Participants

Baseline characteristics of socio-demographic factors, sodium intake, energy intake, and anthropometric measurements are presented in Table 1. This study recruited 3300 children and adolescents with an average age of 12.01 years old, and 28.8% of participants aged 7–9 years old. The sodium intake was 4102 mg/day, and the participants showed average intake of energy, carbohydrates, fat, saturated fatty acids, protein and fiber were 1858 kcal/day, 262 g/day, 68 g/day, 16 g/day, 54 g/day, and 9 g/day. In total, 18.0% children and adolescents were either overweight or obese, and 9.0% were classified as abdominal obese.

### 3.2. Anthropometric Measures and Key Characteristics by Tertile of Sodium Intake

Table 2 shows anthropometric measures and key characteristics by tertile of dietary sodium intake. The BMI and consumption of total energy, protein and fat increased with increasing consumption of dietary sodium both in 7–9 years old and 10–18 years old. In both 7–9 years and 10–18 years old groups, the prevalence of overweight/obesity increased with higher intake of sodium.

### 3.3. Anthropometric Measures and Key Characteristics by Tertile of Sodium Density

Table 3 shows anthropometric measures and key characteristics by tertile of sodium density. The consumption of total energy intake and protein declined with increasing consumption of sodium density. BMI was not different across tertile of sodium density, as did the proportion of children and adolescent who were overweight/obesity both in 7–9 years old and 10–18 years old group. In 10–18 years old groups, the prevalence of abdominal obesity increased with higher sodium density.

### 3.4. Association between Dietary Sodium Intake and Overweight/Obesity

Table 4 shows varying degrees of association between dietary sodium intake and the two type of overweight/obesity. The multivariate adjusted OR for overweight/obesity risk assessed by BMI and adiposity risk assessed by WHtR in children and adolescent of the highest tertile of dietary sodium intake in Model 2, comparing with the lowest tertile, were 1.45 (95%CI 1.11–1.88) and 1.75 (95%CI 1.22–2.53). Adding energy intake to the Model 3 did not alter the general pattern of the association described above Model 2. Adding carbohydrates, protein, fat, saturated fatty acids, fiber, and energy intake to the Model 4 did not alter the general pattern of the association described above Model 3. When stratified by age group, after adjusted for age, gender, household income, area, protein, carbohydrates, protein, fat, saturated fatty acids, fiber, and energy intake, subjects aged 10–18 years old in highest of sodium intake had higher OR for overweight/obesity and adiposity compared with those in the lowest tertile for overweight/obesity and abdominal obesity.

### 3.5. Association between Sodium Density and Overweight/Obesity

Table 5 shows varying degrees of association between sodium density and the two type of overweight/obesity. OR of overweight/obesity and abdominal obesity increased significantly across increasing tertile of sodium density among total children and adolescents. The multivariate adjusted OR abdominal obesity risk assessed by WHtR in children and adolescent of the highest tertile of sodium density in Model 2 were higher comparing with the lowest tertile. Adding energy intake to the Model 3, there are higher risk of overweight/obesity and adiposity among children and adolescents of the highest tertile of sodium density comparing with the lowest tertile. Adding carbohydrates, protein, fat, saturated fatty acids, and fiber intake to the Model 4 did not alter the general pattern of the association described above Model 3. When stratified by age group, after adjusted for age, gender, household income, area, carbohydrates, protein, fat, saturated fatty acids, and fiber intake, subjects age 10–18 years old in highest of sodium density had higher OR for overweight/obesity and adiposity compared with those in the lowest tertile for BMI and WHtR.

## 4. Discussion

This study found that higher sodium intake was positive associated with overweight/obesity in Chinese children and adolescents. After adjusting for socio-demographic variables and energy intake the positive relationship between sodium intake and overweight/obesity was founded, the same positive relationship was found between sodium density and overweight/obesity and abdominal obesity. Carbohydrates, protein, fat, saturated fatty acids, and fiber intake were adjusted in Model 4 did not alter relationship between overweight/obesity and sodium intake. We did not find positive relationship between sodium intake and overweight/obesity in the younger age group, which perhaps due to the small sample size in the younger age group. According to this study, high sodium intake was an important risk factor for overweight/obesity among children and adolescent and this effect was independent of energy and macronutrient intake.

In recent years, the study on the effect of dietary sodium on children and adolescent has attracted more and more attention [8,11,15,24,25]. Some studies shown that the risk of obesity increases with sodium intake, which may be due to the increase in sodium intake is accompanied by an increase in energy intake [14,26], such as Libuda et al. presume that the association between high-salt diet and obesity is related to increased intake of high-energy salty foods like cheese [15], Hoffmann et al. reported that obese group consumed more salt along with more food intake [27]. Different from those studies, the present study has shown that sodium intake was associated with overweight/obesity and abdominal obesity independent of daily energy intake among children and adolescents, this finding is in agreement with previously studies which showed that energy intake does not differ between the highest and lowest quartiles of sodium excretion [26,27], and several previous studies carried out in Korea and Australia showed that overweight/obesity related with dietary sodium may exist regardless of energy intake [12,15]. In addition, participants in highest tertiles of sodium density have higher risk of overweight/obesity and abdominal obesity compared with the those in the lowest tertiles of sodium density, those findings are consistent with previous study conducted in Korea; children and adolescents in the highest quintile of sodium density were more likely to be obese or centrally obese [28].

It should be noted that dietary sodium intake among children and adolescent is 4102 mg/day, which exceeded the maximum recommended amount set by WHO [29]. In China, the main source of dietary sodium was salt; a previous study reported that salt accounted for more than two-thirds (68.7%) of total dietary sodium intake [30]. Therefore, reducing salt intake is a key stratagem to prevent overconsumption of sodium intake.

Another possible explanation for the adverse effects of dietary sodium is that a high-sodium consumer might intake more fats, saturated fatty acids, carbohydrates, and protein. In our study, the participants in the highest tertile of dietary sodium consumed more fats, saturated fatty acids, carbohydrates, and protein; however, the participants in the highest tertile of dietary sodium density consumed less fats, saturated fatty acids, carbohydrates, and protein. In order to study this hypothesis, we adjusted potential confounding factor in multiple logistic analyses, but failed to demonstrate this indirect effect. Instead, we found that sodium intake and sodium density was associated with overweight/obesity and abdominal obesity independent of consumption of fat, saturated fatty acids, protein, fiber, and total energy intake. There are several mechanisms that may explain the relationship between dietary sodium intake and overweight/obesity. First, dietary sodium increases the risk of obesity may be associated with addiction to salted food. A prospective study showed that salted food may be an addictive substance that stimulates dopamine receptors in the brain’s reward and pleasure center. Salted food withdrawal stimulates appetite, increases calorie consumption, increases the incidence of overeating and overweight/obesity [31]. Another possible mechanism between sodium intake and obesity would be resulted in higher water retention leading to higher body weight [24]. The effect of over consumption of sodium on obesity has also been verified by animal experiments. When the total energy intake of the two groups of rats is the same, comparing with the normal diet group, the plasma leptin concentration of the rats in the high-sodium diet group changed, showing a stronger ability of glucose uptake, and the conversion rate of transforming it into fat was higher. The fat cells of rats in the high-sodium diet group have a greater increase in fat cells and a larger fat cell volume [17]. It is not clear whether sodium has a similar effect on human glucose metabolism, but a positive correlation between higher 24 h urine sodium and metabolic syndrome has been found in adults [32].

There were three limitations in this study. First, this study was limited by the cross-sectional nature of CNNHS 2010–2012; therefore, temporal relationship between sodium intake and obesity could not be established. Second, the amount of sodium intake contributed by edible oil and ingredients from outside the home could not obtain from the CNNHS 2010–2012. The sodium from eating outside was calculated based on share of energy eaten at home and outside, Du et al. reported that eating outside contributed 15·4% to dietary energy intake in total, which could have led to an underestimation of total dietary sodium intake [33,34]. Although previous study reported that dietary sodium and energy were higher when eating outside than eating at home, the difference was not statistically significant [34,35]. Otherwise, most of the participants in present study are students, and eating at canteen was main place for eating out, dietary sodium provided by dining halls may differ little from that provided at home. For estimating sodium intake, 24 h urinary sodium excretion is the gold standard, but due to the large number of recruited adults in this study, total dietary sodium was estimated from three consecutive days. Finally, the sodium results obtained in this study are higher than recalls combined with the household food weighing method instead of 24 h urinary sodium excretion; thus, the consumption of sodium may have been underestimated.

## 5. Conclusions

In conclusion, this study demonstrated the significant association between dietary sodium intake and adiposity in Chinese children and adolescents, independent of energy intake. Having less dietary sodium intake should be considered to prevent overweight/obesity among children and adolescents.

## Figures and Tables

**Table 1:** Baseline characteristics among Chinese children and adolescents aged 7–18 years.

Variable	Total	7–9 Years	10–18 Years
Participants (*n*)	3300	949 (28.8)	2351 (71.2)
Age (years)	12.01 ± 0.10	8.54 ± 0.03	13.40 ± 0.08
Weight (kg)	39.77 ± 0.48	27.75 ± 0.34	44.61 ± 0.54
Height (cm)	146.34 ± 0.56	129.83 ± 0.36	152.99 ± 0.53
BMI (kg/m^2^) *	18.02 ± 0.12	16.31 ± 0.13	18.72 ± 0.14
Gender (%)			
Boys	1780 (56.5)	521 (55.9)	1259(56.7)
Girls	1520(43.5)	428(44.1)	1092(43.3)
Area (%)			
Rural	1292(38.8)	332(35.7)	960(40.0)
Urban	2008(61.2)	617(64.3)	1391(60.0)
Household income (%) **			
Low	1969(63.8)	558(62.1)	1411(64.5)
Middle	947(29.8)	270(30.3)	677(29.6)
High	225(6.4)	70(7.7)	155(5.9)
Overweight/obese (%)			
No	2684(82.0)	755(79.8)	1929(82.9)
Yes	616(18.0)	194(20.2)	422(17.1)
Abdominal obesity (%)			
No	3014(91.0)	877(92.3)	2137(90.4)
Yes	286(9.0)	72(7.7)	214(9.6)
Sodium intake (mg/day) ^a^	4102 ± 90	3811 ± 101	4219 ± 99
Energy intake (kcal/day) ^a^	1858 ± 31	1658 ± 33	1938 ± 33
Sodium density	2340 ± 60	2419 ± 73	2308 ± 62
Carbohydrates (g/day) ^a^	262 ± 6	230 ± 6	275 ± 6
Fat (g/day) ^a^	68 ± 1	63 ± 2	70 ± 2
Saturated fatty acids(g/day) ^a^	16 ± 1	15 ± 1	16 ± 1
Protein (g/day) ^a^	54 ± 1	48 ± 1	57 ± 1
Fiber(g/day) ^a^	9 ± 0	7 ± 0	9 ± 0

*: Body mass index, **: household income was divided into three levels: low household income (<¥10,000 (€1285)), middle household income (¥10,000–¥30,000 (€1285–€3855)) and high household income (>¥30,000 (€3855)). ^a^: dietary intake of subgroups are significantly different (*p* < 0.05).

**Table 2:** Anthropometric measurements according to tertiles of sodium intake among Chinese children and adolescents aged 7–18 years.

Tertile of Sodium Intake						
Variable	7–9 Years			10–18 Years		
	T1 < 2669.15	T2 2669.15–4212.02	T3 > 4212.02	T1 < 3035.11	T2 3035.11–4703.03	T3 > 4703.03
Participants (*n*)	316(33.3)	317(33.4)	316(33.3)	783(33.3)	784(33.4)	784(33.3)
Age (years)	8.50 ± 0.05	8.59 ± 0.04	8.53 ± 0.05	13.24 ± 0.10	13.20 ± 0.12	13.75 ± 0.10*^a^*
Weight (kg)	26.47 ± 0.38	28.07 ± 0.58	28.7 ± 0.49 *^a^*	42.92 ± 0.75	43.38 ± 0.67	47.4 ± 0.73 *^a^*
Height (cm)	128.59 ± 0.47	130.28 ± 0.64	130.6 ± 0.49 *^a^*	151.29 ± 0.66	152.37 ± 0.77	155.2 ± 0.67 *^a^*
BMI (kg/m^2^)	15.87 ± 0.17	16.33 ± 0.19	16.71 ± 0.21 *^a^*	18.37 ± 0.19	18.36 ± 0.17	19.39 ± 0.21 *^a^*
**Gender (%)**						
Boys	168(55.4)	170(54.1)	183(58.4)	375(51.0)	424(57.5)	460(61.2) *^b^*
Girls	148(44.6)	147(45.9)	133(41.6)	408(49.0)	360(42.5)	324(38.8)
**Area (%)**						
Rural	87(27.9)	115(37.8)	130(41.4) *^b^*	310(38.4)	331(42.2)	319(39.4)
Urban	229(72.1)	202(62.2)	186(58.6)	473(61.6)	453(57.8)	465(60.6)
**Household Income (%)**						
Low	193(65.6)	183(62.2)	182(58.5)	486(66.7)	460(63.4)	465(63.3)
Middle	83(28.0)	87(28.3)	100(34.4)	209(28.5)	235(30.0)	233(30.4)
High	19(6.4)	27(9.5)	24(7.1)	50(4.8)	56(6.6	49(6.3)
**Overweight/Obese (%)**						
No	260(82.3)	258(82.8)	237(74.3) *^b^*	658(84.4)	652(85.1)	619(79.3) *^b^*
Yes	56(17.7)	59(17.2)	79(25.7)	125(15.6)	132(14.9)	165(20.7)
**Abdominal Obesity (%)**						
No	297(93.8)	295(93.3)	285(90.0)	731(92.6)	722(92.3)	684(86.5) *^b^*
Yes	19(6.2)	22(6.7)	31(10.0)	100(7.4)	62(7.7)	100(13.5)
Sodium intake (mg/day)	1960 ± 34	3368 ± 25	6067 ± 116 *^c^*	2130 ± 32	3819 ± 21	6584 ± 79 *^c^*
Energy intake (kcal/day)	1538 ± 46	1635 ± 42	1797 ± 42 *^c^*	1727 ± 36	1924 ± 43	2153 ± 39 *^c^*
Carbohydrates (g/day)	225 ± 8	226 ± 8	237 ± 6	257 ± 7	276 ± 8	290 ± 7 *^c^*
Fat (g/day)	53 ± 2	61 ± 2	73 ± 3 *^c^*	57 ± 1	68 ± 2	85 ± 2 *^c^*
Saturated fatty acids (g/day)	13 ± 1	14 ± 1	16 ± 1	14 ± 1	15 ± 1	19 ± 1
Protein (g/day)	44 ± 1	49 ± 1	51 ± 2 *^c^*	51 ± 1	57 ± 1	62 ± 1 *^c^*
Fiber (g/day)	7 ± 0	7 ± 0	8 ± 0	8 ± 0	9 ± 0	10 ± 0
Sodium density (mg/kcal)	1397 ± 44	2201 ± 52	3637 ± 104 *^c^*	1354 ± 32	2191 ± 42	3325 ± 68 *^c^*

T: Tertile; BMI: body mass index; data of categorical variables expressed as number (%); *^a^* ANOVA for continuing variable, *p* < 0.05; *^b^* Rao–Scott test for categorized variables, *p* < 0.05; *^c^* Kruskal–Wallis test for skewed distribution variables, *p* < 0.05.

**Table 3:** Anthropometric measurements according to tertiles of sodium density among Chinese children and adolescents aged 7–18 years.

Variable	Tertile of Sodium Density					
	7–9 Years			10–18 Years		
	T1 < 1753.16	T2 1753.16–4212.02	T3 > 2574.79	T1 < 1674.22	T2 1674.22–2570.79	T3 > 2570.79
Participants (*n*)	317(33.4)	316(33.3)	316 (33.3)	784 (33.4)	783(33.3)	784(33.3)
Age (years)	8.57 ± 0.05	8.50 ± 0.05	8.55 ± 0.05	13.51 ± 0.11	13.29 ± 0.11	13.41 ± 0.11
Weight (kg)	27.13 ± 0.44	28.09 ± 0.52	28.01 ± 0.49 *^a^*	43.9 ± 0.76	44.62 ± 0.76	45.27 ± 0.7
Height (cm)	129.25 ± 0.56	129.98 ± 0.57	130.24 ± 0.47	152.49 ± 0.72	153.19 ± 0.73	153.27 ± 0.67
BMI (kg/m^2^)	16.04 ± 0.18	16.45 ± 0.19	16.41 ± 0.22	18.5 ± 0.19	18.63 ± 0.18	19 ± 0.22
**Gender (%)**						
Boys	180(59.0)	174(56.0)	167(53.0)	420(57.0)	433(58.4)	406(54.7)
Girls	137(41.0	142(44.0)	149(47.0)	364(43.0)	350(41.6)	378(45.3)
**Area (%)**						
Rural	76(23.0)	129(42.1)	127(41.5) *^b^*	272(31.9)	338(42.4)	350(45.4) *^b^*
Urban	241(77.0)	187(57.9)	189(58.5)	512(68.1)	445(57.6)	434(54.6)
**Household Income (%)**						
Low	195(65.3)	174(58.9)	189(62.0)	471(65.1)	453(63.1	487(65.2)
Middle	86(28.8)	99(33.7)	85(28.5)	219(29.6)	245(31.3)	213(28.1)
High	17(5.9)	19(7.4)	34(9.5)	55(5.2)	51(5.7)	49(6.7)
**Overweight/Obese (%)**						
No	252(79.6)	251(80.7)	252(79.2)	660(85.3)	636(82.1)	633(81.4)
Yes	65(20.4)	65(19.3)	64(20.8)	124(14.7)	147(17.9)	151(18.6)
**Abdominal Obesity (%)**						
No	295(92.4)	293(93.4)	289(91.2)	731(92.5)	711(90.8)	695(88.2) *^b^*
Yes	22(7.6)	23(6.6)	27(8.8)	53(7.5)	72(9.2)	89(11.8)
Sodium intake (mg/day)	2244 ± 73	3402 ± 68	5715 ± 145 *^c^*	2479 ± 66	3994 ± 60	6064 ± 108 *^c^*
Energy intake (kcal/day)	1868 ± 56	1592 ± 33	1522 ± 35 *^c^*	2198 ± 59	1913 ± 29	1719 ± 27 *^c^*
Carbohydrates (g/day)	272 ± 11	218 ± 5	200 ± 5	329 ± 11	268 ± 5	230 ± 5
Protein (g/day)	54 ± 2	47 ± 1	44 ± 1 *^c^*	63 ± 1	57 ± 1	51 ± 1 *^c^*
Fat (g/day)	65 ± 2	61 ± 2	62 ± 2 *^c^*	72 ± 2	70 ± 2	68 ± 2 *^c^*
Saturated fatty acids (g/day)	16 ± 1	14 ± 1	13 ± 1	18 ± 1	16 ± 1	14 ± 1
Fiber (g/day)	8 ± 0	7 ± 0	7 ± 0	10 ± 0	9 ± 0	9 ± 0

T: Tertile; BMI: body mass index; data of categorical variables expressed as number (%); *^a^* ANOVA for continuing variable, *p* < 0.05; *^b^* Rao–Scott test for categorized variables, *p* < 0.05; *^c^* Kruskal–Wallis test for skewed distribution variables, *p* < 0.05.

**Table 4:** Odds ratios for overweight/obesity and abdominal obesity according to tertiles of sodium intake among Chinese children and adolescents aged 7–18 years.

Models	Total			7–9 Years			10–18 Years		
	T1	T2	T3	T1	T2	T3	T1	T2	T3
**Overweight/Obesity**									
Model 1	1	0.95(0.77–1.18)	1.48(1.13–1.94)	1	0.97(0.61–1.53)	1.62(1.05–2.49)	1	0.95(0.72–1.25)	1.42(1.03–1.95)
Model 2	1	0.90(0.72–1.13)	1.45(1.11–1.88)	1	0.91(0.56–1.47)	1.42(0.93–2.17)	1	0.88(0.66–1.19)	1.52(1.09–2.12)
Model 3	1	0.86(0.68–1.09)	1.33(1.00–1.76)	1	0.85(0.52–1.39)	1.19(0.77–1.84)	1	0.87(0.65–1.16)	1.46(1.03–2.06)
Model 4	1	0.86(0.68–1.09)	1.30(1.00–1.75)	1	0.87(0.54–1.43)	1.26(0.82–1.93)	1	0.86(0.64–1.16)	1.44(1.02–2.05)
**Abdominal Obesity**									
Model 1	1	1.06(0.74–1.53)	1.89(1.33–2.67)	1	1.08(0.57–2.06)	1.67(0.84–3.35)	1	1.06(0.71–1.57)	1.96(1.32–2.91)
Model 2	1	0.95(0.65–1.40)	1.75(1.22–2.53)	1	1.02(0.52–1.98)	1.39(0.66–2.96)	1	0.94(0.62–1.42)	1.92(1.26–2.91)
Model 3	1	0.94(0.63–1.38)	1.69(1.12–2.56)	1	0.99(0.50–1.93)	1.19(0.53–2.64)	1	0.95(0.62–1.44)	1.95(1.23–3.09)
Model 4	1	1.01(0.73–1.40)	1.70(1.19–2.44)	1	0.83(0.46–1.51)	0.91(0.45–1.64)	1	1.08(0.75–1.56)	2.14(1.34–3.42)

Model 1: unadjusted, Model 2: adjusted for gender, age, household income, and area, Model 3: adjusted for gender, age, household income, area, and energy intake, Model 4: adjusted for gender, age, household income, area, energy, carbohydrates, protein, fat, saturated fatty acids, and fiber intake.

**Table 5:** Odds ratios for overweight/obesity and abdominal obesity according to tertiles of sodium density among Chinese children and adolescents aged 7–18 years.

Models	Total			7–9 Years			10–18 Years		
	T1	T2	T3	T1	T2	T3	T1	T2	T3
**Overweight/Obesity**									
Model 1	1.00	1.14 (0.86–1.52)	1.22 (0.90–1.65)	1.00	0.93 (0.63–1.39)	1.02 (0.65–1.62)	1.00	1.26 (0.91–1.75)	1.32 (0.93–1.87)
Model 2	1.00	1.06 (0.78–1.44)	1.20 (0.89–1.64)	1.00	0.85 (0.55–1.32)	0.96 (0.61–1.50)	1.00	1.20 (0.84–1.71)	1.36 (0.96–1.92)
Model 3	1.00	1.17 (0.87–1.59)	1.73 (1.16–2.58)	1.00	1.06 (0.67–1.66)	1.26 (0.79–2.02)	1.00	1.28 (0.90–1.83)	1.51 (1.05–2.17)
Model 4	1.00	1.17 (0.86–1.59)	1.39 (1.03–1.90)	1.00	1.06 (0.67–1.68)	1.28 (0.80–2.06)	1.00	1.26 (0.88–1.80)	1.47 (1.03–2.10)
**Abdominal Obesity**									
Model 1	1.00	1.14 (0.76–1.71)	1.52 (1.04–2.23)	1.00	0.86 (0.45–1.64)	1.18 (0.61–2.28)	1.00	1.26 (0.82–1.94)	1.66 (1.09–2.54)
Model 2	1.00	1.04 (0.69–1.57)	1.53 (1.03–2.27)	1.00	0.66 (0.32–1.39)	1.15 (0.57–2.33)	1.00	1.20 (0.79–1.84)	1.69 (1.10–2.61)
Model 3	1.00	1.12 (0.74–1.70)	1.73 (1.16–2.58)	1.00	0.83 (0.38–1.79)	1.54 (0.72–3.32)	1.00	1.26 (0.82–1.94)	1.84 (1.19–2.85)
Model 4	1.00	1.35 (0.94–1.93)	1.84 (1.31–2.59)	1.00	1.13 (0.66–1.94)	1.07 (0.53–2.17)	1.00	1.46 (0.95–2.266)	2.24 (1.48–3.39)

Model 1: unadjusted, Model 2: adjusted for gender, age, household income, and area, Model 3: adjusted for gender, age, household income, area, and energy intake, Model 4: adjusted for gender, age, household income, area, energy, carbohydrates, protein, fat, saturated fatty acids, and fiber intake.

## Data Availability

Sorry, the data supporting reported results of present manuscript is non-public.
